# Comparative Chloroplast Genomics Reveals Intrageneric Divergence in *Salix*

**DOI:** 10.3390/ijms26052248

**Published:** 2025-03-03

**Authors:** Fulin Yuan, Liwei Zhou, Xueya Wei, Ce Shang, Zhixiang Zhang

**Affiliations:** School of Ecology and Nature Conservation, Beijing Forestry University, Beijing 100083, China; yfl2020@bjfu.edu.cn (F.Y.); zlw7676@bjfu.edu.cn (L.Z.); weixueya@bjfu.edu.cn (X.W.)

**Keywords:** phylogeny, chloroplast genome, genome comparative analysis, simple sequence repeats, molecular evolution

## Abstract

As the most diverse genus of Salicaceae, *Salix* is primarily distributed in the temperate zone of the Northern Hemisphere, encompassing 350–500 species worldwide. The genus’s evolutionary history is complex due to significant genetic differentiation. Chloroplast genes, being highly conserved, serve as effective tools for studying uniparental inheritance and evolution. In this study, we sequenced and assembled the chloroplast genomes of five representative *Salix* species. Phylogenetic relationships were constructed using chloroplast genome data, and structural differences among lineages were compared. These *Salix* chloroplast genomes exhibited a typical quadripartite structure, with lengths ranging from 154,444 to 155,725 bp. We successfully annotated 131 genes, including 88 protein-coding genes, 35 tRNA genes, and 8 rRNA genes. Clade I showed higher variability in the SSC region, identifying five highly variable regions: *petA-psbJ*, *rps16-rps3*, *ndhD*, *ccsA-ndhD*, and *ndhG-ndhI*. Two rapidly evolving genes, *ndhI* and *ycf4*, were also identified. The total length of insertions and deletions (InDels) in Clade I was 1046 bp. Clade II exhibited greater variability in the LSC region, with four highly variable regions being identified: *trnK-trnQ*, *ndhC-trnV*, *trnV*, and *psdE-petL*. Four rapidly evolving genes—*infA*, *rpoC1*, *rps18*, and *ycf1*—were identified. The total length of InDels in Clade II was 1282 bp. Therefore, this study elucidated the chloroplast genome evolution across different *Salix* lineages, thereby providing deeper insights into intrageneric phylogenetic relationships.

## 1. Introduction

*Salix*, the largest genus in the Salicaceae family, comprises approximately 350 to 500 species worldwide [1]. It is widely distributed across temperate and frigid zones of the Northern Hemisphere. Extensive hybridization and genetic introgression among species have resulted in high genetic diversity and a complex evolutionary history, making Salix an excellent model for studying plant genetic evolution [2,3,4].

*Salix* classification commonly relies on subgenera and sections. Based on the morphology of American willows, two subgenera—subg. *Salix* and subg. *Vetrix*—were initially accepted [5]. Argus conducted a cluster analysis of phenotypic traits and divided 127 New World willow species into four subgenera: subg. *Salix*, subg. *Longifoliae*, subg. *Chamaetia*, and subg. *Vetrix*. Subsequently, North American *Salix* was reclassified into five subgenera: *Salix*, *Protitea*, *Longifoliae*, *Chamaetia*, and *Vetrix* [6,7]. Advancements in bioinformatics have enabled the study of species evolution using genomic data, providing insights into true evolutionary relationships through molecular systematics [8]. Molecular phylogenetic studies have shown that *Salix* is monophyletic, forming two distinct lineages with inconsistent internal topology. Wu (2015) analyzed the nuclear ribosomal DNA external transcribed spacer (ETS), the internal transcribed spacer (ITS), and four plastid markers, classifying *Salix* into two clades: one comprising subgenera *Salix*, *Longifoliae*, and *Protitea*, and the other including subg. *Chosenia*, *Pleuradenia*, *Chamaetia*, and *Vetrix*. That study also supported the merging of *Chamaetia* and *Vetrix* [9]. Zhang (2018) used whole chloroplast genome sequences from 42 Salicaceae species to estimate divergence times, confirming the division of *Salix* into two main clades: the *Chamaetia*/*Vetrix* clade and the *Salix* clade [10]. Sergey Gulyaev (2022) employed whole-genome resequencing data and classified *Salix* into two major groups: the paraphyletic *Salix* grade and the *Vetrix* clade [11]. These inconsistencies in phylogenetic relationships highlight the complex evolutionary history and high genetic divergence of *Salix*.

Chloroplast genomes are widely used in genetic evolution studies due to their relatively conserved structure, abundant genetic information, moderate base substitution rate, and distinct differences in molecular evolution rates between coding and non-coding regions [12]. The LSC and SSC regions typically exhibit higher variation because they contain more protein-coding genes and non-coding intergenic sequences, particularly functional genes related to photosynthesis and energy conversion, which are more susceptible to selection pressure [13]. The *matK*, *rbcL*, *psbA*, *ndhC*, *ndhK*, and *accD* genes in the LSC region, along with *ndhF*, *ycf1*, and *ndhI* in the SSC region, have been identified as rapidly evolving chloroplast genes [14,15]. Comparative studies of chloroplast genomes in *Salix* have shown that sampling methods significantly influence results. For example, an analysis of the chloroplast genome characteristics and interspecific variation of three shrub willow species revealed high similarity in their chloroplast genomes [16]. Another study identified three sand-fixing shrub willow species and found greater variation in non-coding and intergenic regions than in coding regions [17]. Zhou (2021) compared chloroplast genomes from 21 *Salix* species and identified seven positively selected genes, with different gene types in trees and shrubs [18]. Most research on *Salix* chloroplast genomes has focused on one or a few species, often using comparative analyses of randomly collected samples. However, this approach does not fully capture variation patterns and evolutionary trends across the genus.

To address this, we developed a comprehensive framework by extensively sampling *Salix* species and constructing a chloroplast genome-based phylogenetic tree. This tree was then used to examine structural differences among clades, providing deeper insights into *Salix* evolution from the perspective of maternal inheritance.

## 2. Results

### 2.1. Phylogenetic Relationships in Salix

**Figure 1:**
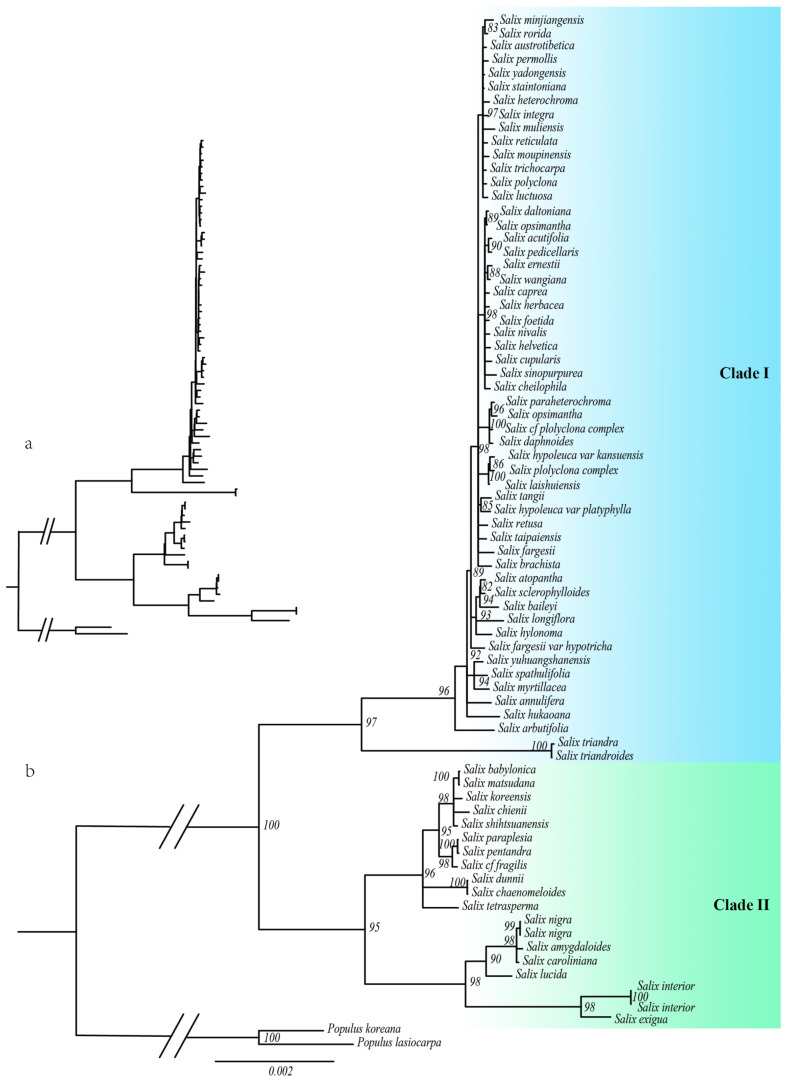
The phylogenetic tree of the chloroplast genome was constructed using the maximum likelihood (ML) method. (**a**) Chloroplast phylogenetic structure map before the bootstrap support (BS) merger. (**b**) The phylogenetic tree. (Merge branches with less than 80 support).

**Figure 2:**
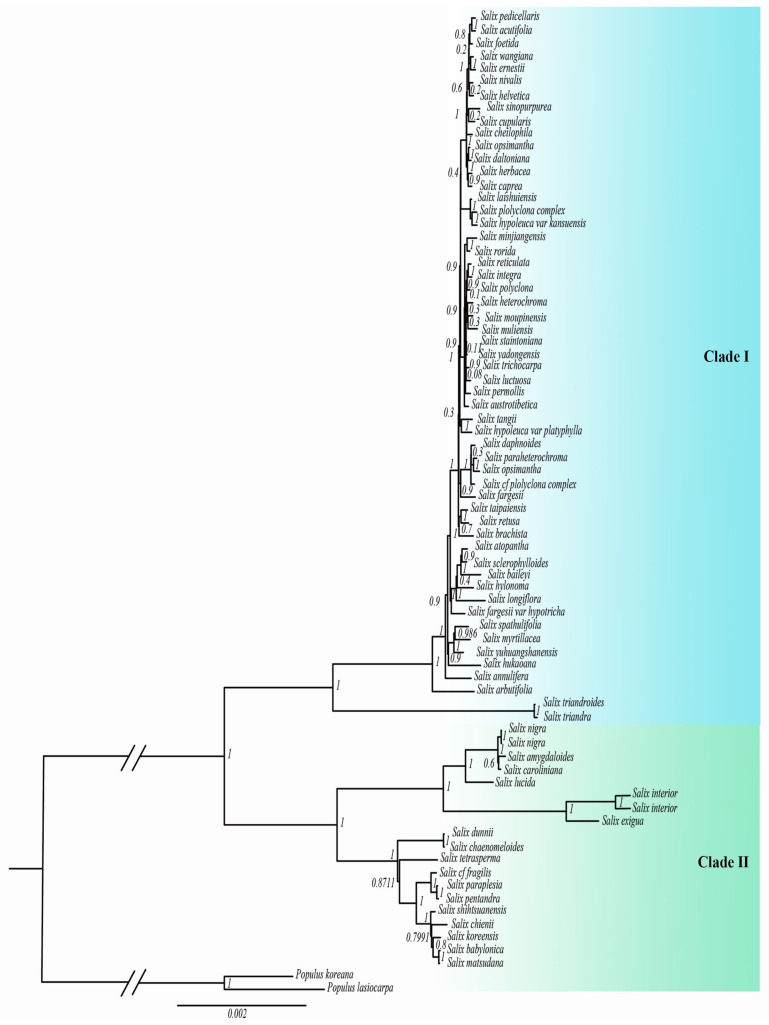
The phylogenetic tree of the chloroplast genome was constructed using Bayesian inference (BI).

Both maximum likelihood (ML) and Bayesian inference (BI) confirmed that *Salix* is a strongly monophyletic group, although internal branch support was relatively low. This may be due to hybridization and recent radiation in *Salix*. The phylogenetic tree classified *Salix* into two main clades, Clade I and Clade II, consistent with previous studies (Figure 1 and Figure 2). Clade I includes species from subg. *Chamaetia* and subg. *Vetrix*, while Clade II includes species from subg. *Salix*, subg. *Protitea*, and subg. *Longifoliae*. In Clade I, the ML and BI trees showed notable inconsistencies, whereas the matrilineal genetic relationships in Clade II were well resolved.

The chloroplast phylogeny of Salix varied considerably across lineages, indicating divergent evolutionary trajectories of the chloroplast genome. To investigate these differences, we selected representative species from each lineage for comparative genomic analysis. In Clade I, *Salix integra*, *Salix pedicellaris*, *Salix opsimantha*, *Salix laishuiensis*, *Salix tangii*, *Salix taipaiensis*, *Salix longiflora*, *Salix myrtillacea*, *Salix arbutifolia*, and *Salix triandra* were analyzed. In Clade II, *Salix babylonica*, *Salix dunnii*, *Salix tetrasperma*, *Salix nigra*, *Salix lucida*, and *Salix interior* were analyzed.

### 2.2. Chloroplast Genome Features

**Figure 3:**
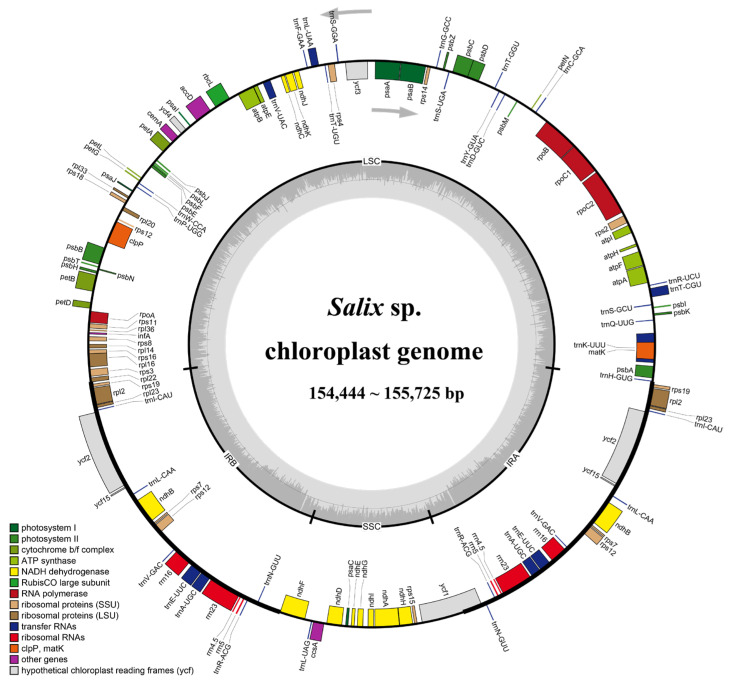
Chloroplast genome gene map of *Salix* sp. Genes located outside the ring are transcribed in a counterclockwise direction, while genes located inside the ring are transcribed in a clockwise direction. The dark gray area in the inner circle is the GC content of the cp genome, and the light gray area is the AT content. Different color blocks represent genes from different functional groups.

The chloroplast genomes of 16 *Salix* species exhibited a typical quadripartite structure, ranging from 154,444 to 155,725 bp in length (Figure 3). A total of 131 genes were annotated across the 16 *Salix* chloroplast genomes, including 88 protein-coding genes, 35 tRNA genes, and 8 rRNA genes. Detailed information is provided in Table S1. These genes were associated with photosynthesis, replication, and other functions. Notably, 18 genes—7 protein-coding genes (*ndhB*, *rpl2*, *rpl23*, *rps12*, *rps19*, *rps7*, and *ycf2*), 4 rRNA genes, and 7 tRNA genes—were duplicated in the inverted repeat regions.

When comparing chloroplast genome features between Clade I and Clade II, Clade I genomes ranged from 154,646 to 155,692 bp in size, whereas Clade II genomes exhibited greater variation, ranging from 154,444 to 155,725 bp. This increased variation in Clade II may have resulted from gene deletions, pseudogene formation, or non-coding region expansion across different species or individuals. The predicted GC content of a genome significantly influences genomic function and species ecology, serving as a key indicator of species affinity [19]. In 16 *Salix* species, chloroplast GC content ranged from 36.64% to 36.78% (Table 1). Although this variation is minimal, it may suggest that *Salix* species underwent a relatively short or slow evolutionary differentiation process.

### 2.3. Comparative Analysis of Salix Chloroplast Genome

**Figure 4:**
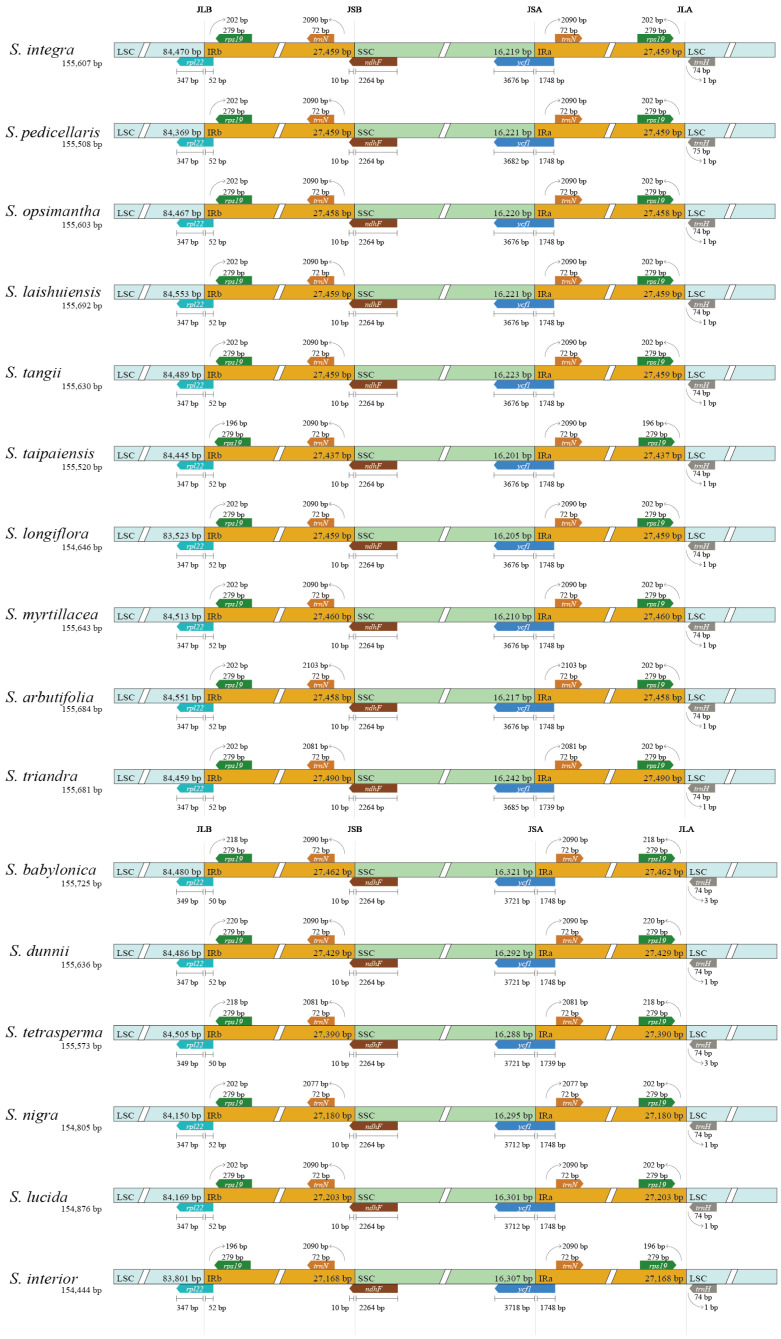
Comparison of junctions between LSC (light blue), SSC (light green), and IR (orange) regions among ten *Salix* species. Distance in figure is not to scale.

Boundary comparisons of the LSC, SSC, and IR regions using IRscope revealed high conservation of gene content at these boundaries, with minimal variation in gene length and positioning among the 16 species. For example, *rpl22* and *rps19* were consistently located at the LSC/IRb boundary, while *trnN* and *ndhF* were found at the IRb/SSC boundary across all genomes analyzed. The SSC/IRa boundary contained *ycf1* and *trnN*, while the IRa/LSC boundary contained *rps19* and *trnH*. Notably, *rpl22* at the LSC/IRb boundary exhibited only a 2 bp variation, *ycf1* at the SSC/IRa boundary varied by 45 bp, and *rps19* at the IRa/LSC boundary differed by 22 bp (Figure 4).

**Figure 5:**
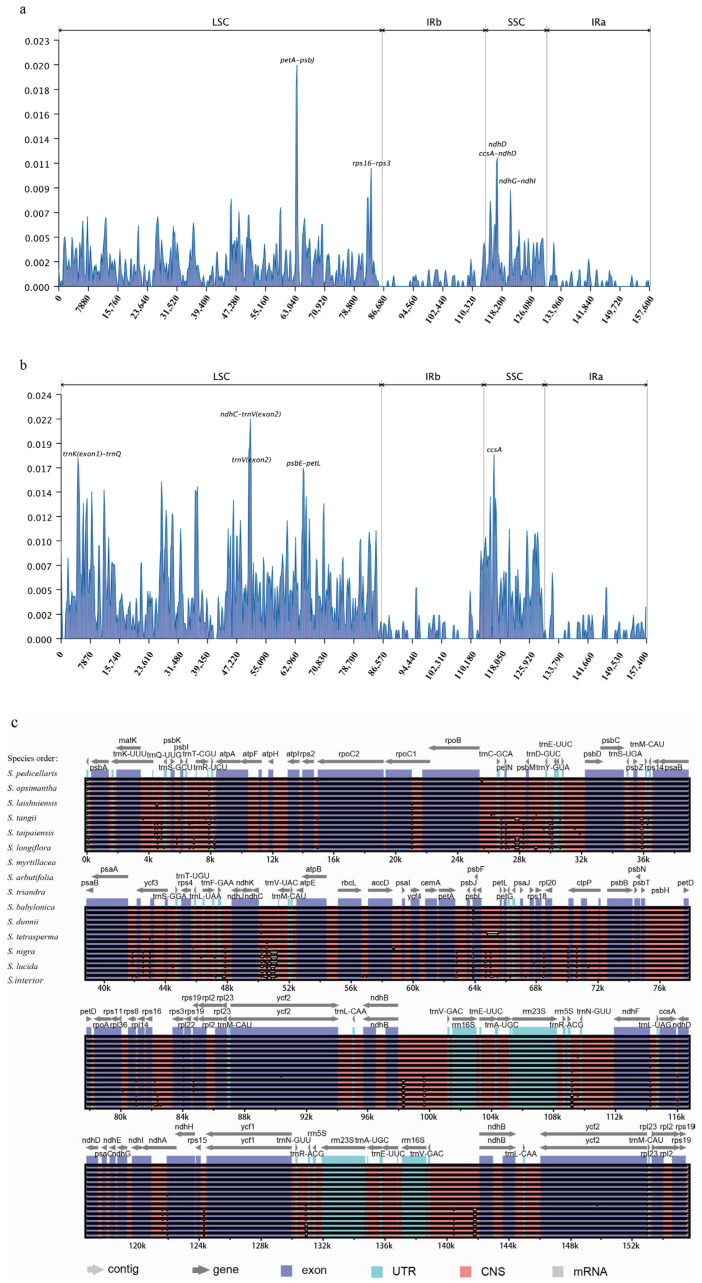
(**a**) Analysis of chloroplast genome nucleotide variability values (Pi) among Clade I. (**b**) Analysis of chloroplast genome nucleotide variability values (Pi) among Clade II. (**c**) Similarity map of chloroplast genome sequence of 16 *Salix* chloroplast genomes created using mVISTA. On the *Y*-axis, the percentage of sequence consistency is shown to be between 50% and 100%. The *X*-axis represents the coordinates of the chloroplast genome. Genomic regions are color-coded as protein-coding (exons), mRNAs or rRNAs, and intergenic regions. Genes are represented by gray arrows.

Genomic variability among the 16 sequenced chloroplast genomes was assessed using mVISTA, with *Salix integra* as the reference. No major genome rearrangements or inversions were detected. Compared with the highly conserved coding regions, non-coding regions (intergenic spacers and introns) exhibited greater variation (Figure 5c). Sliding window analysis using DnaSP v6.0 identified highly variable regions in the SSC region of Clade I, including *petA-psbJ*, *rps16-rps3*, *ndhD*, *ccsA-ndhD*, and *ndhG-ndhI* (Figure 5a). In Clade II, greater variation was observed in the LSC region, particularly in *trnK*-*trnQ*, *ndhC-trnV*, *trnV*, and *psbE-petL* (Figure 5b).

### 2.4. Result of Chloroplast Genome Repeat Sequences

**Figure 6:**
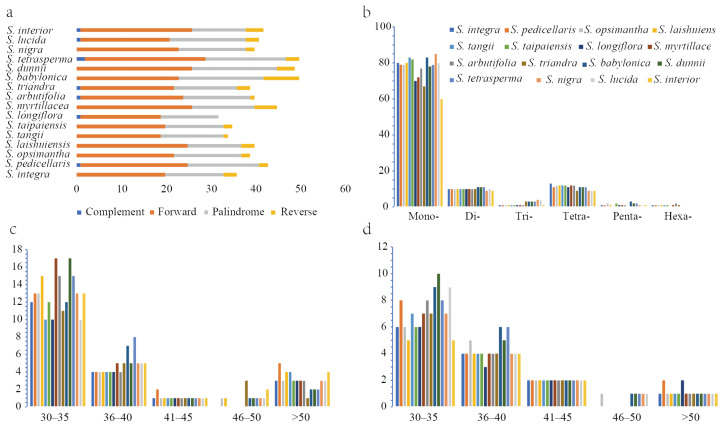
Numbers and types of dispersed repeats of ten *Salix* species in the chloroplast genome. (**a**) Numbers and types of long repeats. (**b**) Numbers and types of SSRs. (**c**) Lengths and frequencies of forward long repeats. (**d**) Lengths and frequencies of palindromic long repeats.

In Clade I, each species contained 90–108 simple sequence repeats (SSRs), with mononucleotide repeats being the most abundant, comprising 74.23% (*Salix myrtillacea*) to 77.57% (*Salix tangii*) of the total (Figure 6b). Each species had 32–45 long repeat sequences, with forward repeats being the most common, followed by palindromic and reverse repeats (Figure 6a). In Clade II, species contained 80–111 SSRs, with mononucleotide repeats accounting for 74.29% (*Salix dunnii*) to 78.7% (*Salix nigra*) (Figure 6b). Long repeat sequences ranged from 41 to 50 per species, with forward repeats being the most common, followed by palindromic and reverse repeats (Figure 6a). Forward and palindromic repeats were predominantly 30–35 bp in length (Figure 6c,d). Detailed information is provided in Tables S3 and S4.

### 2.5. Result of InDel and Selection Pressure

**Figure 7:**
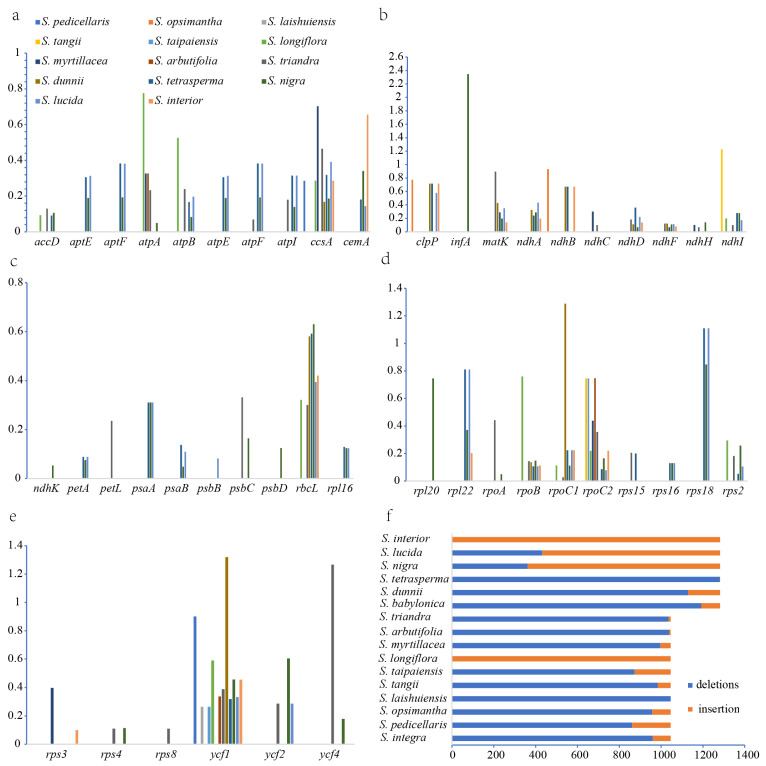
(**a**–**e**) Ka/Ks ratios for 46 protein-coding genes from ten *Salix* species. (**f**) InDels analysis results for *Salix*.

Insertion and deletion (InDel) variations were identified in chloroplast genomes. In Clade I, deletion sites ranged from 0 to 1046 bp and insertion sites from 0 to 1046 bp, with a total InDel variation length of 1046 bp. In Clade II, deletion sites ranged from 1 to 1282 bp and insertion sites from 0 to 1281 bp, with a total InDel variation length of 1282 bp. Clade II exhibited more rapid species divergence (Figure 7f). Ka/Ks ratios were used to assess selection pressure on protein-coding genes in *Salix*. In Clade I, 29 genes were identified, with Ka/Ks ratios ranging from 0.027 to 1.26. The Ka/Ks ratios of *ndhI* and *ycf4* exceeded 1, indicating positive selection. These genes are primarily associated with protease synthesis, photosynthesis, and self-replication. In Clade II, 45 genes were identified, with Ka/Ks ratios ranging from 0.048 to 2.34. Four genes (*infA*, *rpoC1*, *rps18*, and *ycf1*) had Ka/Ks ratios above 1 (Figure 7a–e), primarily related to self-replication. These findings suggest that protein-coding genes in the two clades are undergoing distinct evolutionary processes under different selective pressures.

## 3. Discussion

### 3.1. Phylogenetic Relationships Among the Salix

Phylogenetic analysis based on chloroplast genomes classified *Salix* into two main clades: Clade I and Clade II. Our findings align with previous molecular phylogenetic studies [8,20], supporting Gulyaev [10], but not Ogutcen [21]. Ogutcen classified *Salix* into subg. *Chamaetia*/*Vetrix* and subg. *Salix s.l*., while Chen proposed five subgenera: subg. *Salix*, subg. *Urbaniana*, subg. *Triandrae*, subg. *Longifoliae*, and subg. *Vetrix* [22]. Our study strongly supports the monophyly of subg. *Chamaetia*/*Vetrix*, subg. *Longifoliae*, and subg. *Protitea*.

Phylogenetic trees inferred using maximum likelihood and Bayesian methods also support two *Salix* lineages. However, Clade I exhibits topological inconsistencies between the two methods and low internal branch support. This weak support may stem from two main factors: (1) chloroplast genomes, being more conserved than nuclear genomes, have fewer sequence variations and a limited phylogenetic signal, reducing branch support; and (2) frequent hybridization within the genus has led to chloroplast capture.

Reticulate evolution driven by hybridization, introgression, and lineage sorting may explain the observed molecular phylogenetic incongruence [23]. Hybridization can create novel gene combinations, enhancing progeny adaptability to diverse environments and promoting genetic diversity [24,25]. During frequent hybridization, nuclear genes may follow distinct evolutionary trajectories, leading to highly variable regions. Given the complexity of *Salix* phylogeny, chloroplast data alone are insufficient to resolve its internal topological structure [26,27]. Our results reflect only the evolutionary relationships inferred from chloroplast genomes. Future studies should incorporate nuclear genetic data on parental inheritance and broader species sampling to achieve a more comprehensive understanding of *Salix* phylogeny.

### 3.2. Chloroplast Genome Differences Between Two Lineages of Salix

The structure and variation of *Salix* chloroplast genomes were compared, revealing significant differences between Clades I and II. In Clade II, North American species formed a subclade, while East Asian species formed another. Overall, chloroplast variation was greater in Clade II, likely due to (1) genetic differentiation driven by geographic isolation, leading to the accumulation of chloroplast genome variations; (2) historical biogeographic events, such as glacial isolation, which trapped species in refugia, followed by post-glacial dispersal, resulting in unique genomic features; and (3) chloroplast capture through hybridization between North American and East Asian species.

Nucleotide diversity analysis indicated that IR regions were more conserved than LSC and SSC regions, likely due to sequence homogenization via gene conversion and the role of IRs in genomic stability [28,29]. In Clade I, the SSC region exhibited the highest nucleotide diversity, whereas in Clade II, the LSC region showed the highest diversity. Whether these highly divergent regions or genes can serve as phylogenetic markers for *Salix* species or as molecular markers in population genetics warrants further investigation.

SSRs in chloroplast genomes serve as important molecular markers due to their abundance and high polymorphism, and have been widely used in studies on genetic diversity, species evolution, and conservation biology [30,31]. In both Clade I and Clade II, SSRs and long repeats showed no significant differences, with A/T mononucleotide repeats being dominant, a common feature in other plant lineages [32]. The identified long repeats, particularly forward and palindromic repeats, suggest a role in promoting structural variation and plastid stability. Insertions and deletions (InDels) represent key genomic variations that influence genome size and, consequently, species evolution [33]. To assess structural changes in *Salix* chloroplast genomes during evolution, we calculated the total InDel length for Clade I and Clade II. The InDel length was 1046 bp in Clade I and 1282 bp in Clade II. The significantly greater structural variation in Clade II chloroplast genomes suggests a higher evolutionary rate in Clade II species.

The Ka/Ks ratio is linked to gene adaptive evolution. In plant genomes, nonsynonymous substitutions alter amino acids, potentially modifying protein function and enabling species to adapt to new habitats, whereas synonymous substitutions do not affect protein composition [34]. The Ka/Ks ratio is used to infer gene selection types. Most plant genes have Ka/Ks values below 1, as nonsynonymous substitutions are generally disadvantageous, with only a few genes undergoing positive selection [35,36,37]. A Ka/Ks ratio > 1 indicates rapid positive selection in response to current environmental conditions, playing a crucial role in species adaptation. In Clade I, 29 genes were identified, with the Ka/Ks ratios of *ndhI* and *ycf4* exceeding 1. In Clade II, 45 genes were identified, and 4 genes (*infA*, *rpoC1*, *rps18*, and *ycf1*) had Ka/Ks ratios above 1. The higher number of genes with evolutionary signals in Clade II may be due to its species occupying more ecological niches or facing more complex environments, necessitating greater adaptability. Therefore, their genomes may have experienced stronger environmental selection, a key driver of species diversification and expansion.

## 4. Materials and Methods

### 4.1. Plant Material, DNA Extraction, and Genome Sequencing

Following Argus’s five-subgenus classification [6,7], five *Salix* species were newly sequenced for this study, while 71 species were retrieved from the NCBI GenBank. The dataset included subg. *Salix*, subg. *Protitea*, subg. *Longifoliae*, subg. *Chamaetia*, and subg. *Vetrix*. Detailed species information is provided in Table S2. Genomic DNA was extracted from leaf samples using a modified CTAB method [38]. DNA quality and concentration were assessed with a Qubit 3.0 fluorometer (Thermo Scientific, Waltham, MA, USA) and a NanoDrop 2000c spectrophotometer (NanoDrop Technologies, Wilmington, DE, USA). Qualified DNA samples were fragmented, and sequencing libraries (insert size: 350 bp) were prepared. Sequencing on the Illumina NovaSeq X platform (Illumina, San Diego, CA, USA) generated 5 Gb of data per sample. 

### 4.2. Chloroplast Genome Assembly and Annotation

Raw sequencing reads were quality-filtered using Trimmomatic v0.39. Clean, high-quality reads were used for de novo chloroplast genome assembly with GetOrganelle v1.7.5 [39]. Incomplete assemblies were manually refined in Geneious Prime v2020 [40]. Chloroplast genome annotation was performed with Plastid Genome Annotator Version 3 (PGA) [41] using *Salix annulifera* (GenBank: MZ365447) and *Salix foetida* (GenBank: MZ435429) as references. Manual corrections were made in Geneious Prime, and the final chloroplast genome map was generated using the OGDRAW online tool [42].

### 4.3. Phylogenetic Analysis

Phylogenetic relationships of *Salix* were reconstructed using complete chloroplast genome sequences. *Populus koreana* (GenBank: MW376789) and *Populus lasiocarpa* (GenBank: KX641589) served as outgroups. Detailed species data are provided in Table S2. Sequence alignment was performed using MAFFT with default parameters [43], followed by filtering of unreliable regions with trimAl v1.4.1 [44]. The optimal substitution model was determined using ModelFinder v1.5.4. Phylogenetic analyses were conducted using two methods: maximum likelihood (ML) and Bayesian inference (BI). The ML tree was constructed with RAxML v7.4.2 under the GTRGAMMA model with 1000 rapid bootstrap replicates. The BI tree was inferred using MrBayes 3.2.6, with four MCMC simulations running simultaneously, sampled every 1000 generations over two million generations. The first 25% of trees were discarded as burn-in, and the remaining trees were used to infer phylogenetic relationships. Tracer v1.7 was used to assess parameter convergence and effective sample size, and posterior probabilities were estimated using a 50% majority-rule consensus tree.

### 4.4. Chloroplast Genome Structural Comparison

Structural comparisons of 16 *Salix* chloroplast genomes were performed using the mVISTA program (https://genome.lbl.gov/vista/mvista/submit.shtml, accessed on 11 February 2025) with the Shuffle-LAGAN model. Boundaries of the LSC, IRb, SSC, and IRa regions were analyzed using IRscope (https://irscope.shinyapps.io/irapp/, accessed on 13 February 2025). Clade I sequences were aligned using MAFFT v7.313, and nucleotide diversity (Pi) was calculated using a sliding window analysis in DnaSP v6.0 with a window size of 600 bp and a step size of 200 bp. The same method was applied to Clade II. After aligning all sequences with MAFFT v7.313, insertion and deletion (InDel) site lengths were determined using R 4.4.0. *Salix integra* was used as the reference sequence for Clade I, while *Salix babylonica* was the reference for Clade II.

### 4.5. Analysis of Repeat Sequences and SSRs

Repeat sequences in chloroplast genomes were analyzed using the REPuter program, identifying four types of long repeats: forward, reverse, palindromic, and complementary. The parameters were set as follows: repeat similarity >90%, minimum repeat size of 30 bp, and a Hamming distance of 3. Simple sequence repeats (SSRs) were identified using MISA (http://pgrc.ipk-gatersleben.de/misa/, accessed on 13 February 2025), and six SSR types (mononucleotide, dinucleotide, trinucleotide, tetranucleotide, pentanucleotide, and hexanucleotide) were analyzed with repeat thresholds of 10, 5, 4, 3, 3, and 3, respectively.

### 4.6. Analysis of Gene Selection Pressure

In Clade I, coding sequences and protein sequences from *Salix integra* were extracted. Protein sequences from other species were compared with those of *Salix integra* using BLASTn (v2.10.1) to identify homologous proteins. Homologous protein sequences were aligned using MAFFT (v7.427), and a Perl script was used to map the aligned protein sequences to the corresponding coding sequences. The aligned coding sequences were then used to calculate Ka/Ks values using Ka/Ks_Calculator2 [45]. In Clade II, *Salix babylonica* was used as the reference, following the same methodology.

## Figures and Tables

**Table 1:** Features of the chloroplast genomes of eight Caragana species.

Species		Species Length (bp)	GC (%)	Number of Genes			
				Coding Genes	tRNA	rRNA	Total
Clade I	*S. integra*	155,607	36.69	88	35	8	131
	*S. pedicellaris*	155,508	36.7	88	35	8	131
	*S. opsimantha*	155,603	36.68	88	35	8	131
	*S. laishuiensis*	155,692	36.68	88	35	8	131
	*S. tangii*	155,630	36.69	88	35	8	131
	*S. taipaiensis*	155,520	36.7	88	35	8	131
	*S. longiflora*	154,646	36.78	88	35	8	131
	*S. myrtillacea*	155,643	36.68	88	35	8	131
	*S. arbutifolia*	155,684	36.68	88	35	8	131
	*S. triandra*	155,681	36.65	88	35	8	131
Clade II	*S. babylonica*	155,725	36.64	88	35	8	131
	*S. dunnii*	155,636	36.65	88	35	8	131
	*S. tetrasperma*	155,573	36.66	88	35	8	131
	*S. nigra*	154,805	36.76	88	35	8	131
	*S. lucida*	154,876	36.76	88	35	8	131
	*S. interior*	154,444	36.78	88	35	8	131

## Data Availability

The data presented in the study are deposited in the Genome Sequence Archive in National Genomics Data Center (https://ngdc.cncb.ac.cn/gsa/), accession number: PRJCA036831.

## Supplementary Materials

The following supporting information can be downloaded at: https://www.mdpi.com/article/10.3390/ijms26052248/s1.
